# Prevalence of Smokefree Home Rules — United States, 1992–1993 and 2010–2011

**Published:** 2014-09-05

**Authors:** Brian A. King, Roshni Patel, Stephen D. Babb

**Affiliations:** 1 Office on Smoking and Health, National Center for Chronic Disease Prevention and Health Promotion, CDC

Exposure to secondhand smoke (SHS) from cigarettes causes an estimated 41,000 deaths among nonsmoking U.S. adults each year and an estimated $5.6 billion annually in lost productivity caused by premature death (1,2). In a 2006 report, the Surgeon General concluded that there is no risk-free level of exposure to SHS (1). Although an increasing proportion of the population is covered by state or local comprehensive smokefree laws that prohibit tobacco smoking in all indoor public places and worksites, including restaurants and bars (3,4), millions of nonsmokers continue to be exposed to SHS in areas not covered by smokefree laws or policies, including homes (5). The home is the primary source of SHS exposure for children and a major source of exposure for nonsmoking adults (1). To assess progress toward increasing the proportion of households with smokefree home rules, CDC analyzed the most recent data from the Tobacco Use Supplement to the Current Population Survey. Households were considered to have a smokefree home rule if all adult respondents aged ≥18 years in the household reported that no one was allowed to smoke anywhere inside the home at any time. The analysis found that the national prevalence of smokefree home rules increased from 43.0% during 1992–1993 to 83.0% during 2010–2011. Over the same period, the national prevalence of smokefree home rules increased from 56.7% to 91.4% among households with no adult cigarette smokers and from 9.6% to 46.1% among households with at least one adult smoker. Enhanced implementation of evidence-based interventions (e.g., comprehensive smokefree laws, voluntary smokefree home rules, smokefree multiunit housing policies, and initiatives to educate the public about the health effects of SHS) is warranted to further reduce SHS exposure in the United States (1,2).

The Current Population Survey is a household survey administered to the civilian, noninstitutionalized population by the U.S. Census Bureau.* Since 1992–1993, the Tobacco Use Supplement to the Current Population Survey (TUS-CPS) has collected national and state data regarding tobacco use and tobacco-related attitudes and policies, including home smoking rules. The TUS-CPS was conducted during 1992–1993 (293,543 respondents), 1995–1996 (247,088), 1998–1999 (239,652), 2000 (167,096), 2001–2002 (249,288), 2003 (249,620), 2006–2007 (237,119), and 2010–2011 (229,456). Eligible household members were interviewed by telephone or in their homes; the sample included persons aged ≥15 years until 2003, and those aged ≥18 years during 2006–2007 and 2010–2011. Response rates ranged from 62% (2006–2007 and 2010–2011) to 72% (1992–1993).†

Each household member aged ≥18 years was asked, “Which statement best describes the rules about smoking inside your home?” The response options were, “No one is allowed to smoke anywhere inside your home,” “Smoking is allowed in some places or at some times inside your home,” and “Smoking is permitted anywhere inside your home.” Households were considered to have a smokefree home rule if all adult respondents aged ≥18 years in the household reported that no one was allowed to smoke anywhere inside the home at any time. Households were considered to have one or more smokers if at least one respondent aged ≥18 years had smoked ≥100 cigarettes in their lifetime and now smoked “everyday” or “some days.” Data were adjusted for nonresponse and weighted using the household supplement self-response weight. To ensure comparability across surveys, analyses were restricted to respondents aged ≥18 years. Households with discrepancies in responses (i.e., one respondent reported a smokefree home rule, and another did not) were excluded (range = 1.8% during 2010–2011 to 6.9% during 1992–1993). Point estimates and 95% confidence intervals were used to describe the prevalence of smokefree home rules overall and by state. Differences between groups were assessed using chi-square tests, and logistic regression was used to assess temporal trends (Wald test; p<0.05).

What is already known on this topic?

The U.S. Surgeon General has concluded that there is no risk-free level of exposure to secondhand smoke. Although an increasing proportion of the population is protected by state or local comprehensive smokefree laws that prohibit smoking in all indoor areas of public places and worksites, millions of nonsmokers remain susceptible to secondhand smoke exposure in areas not covered by smokefree laws or policies, including homes.

What is added by this report?

The national prevalence of smokefree home rules increased significantly over the past 2 decades, from 43.0% during 1992–1993 to 83.0% during 2010–2011. During this period, the national prevalence of such rules increased from 56.7% to 91.4% among households with no adult smoker, and from 9.6% to 46.1% among households with at least one smoker.

What are the implications for public health practice?

Although the percentage of households with smokefree home rules has increased considerably since 1992–1993, by 2010–2011 fewer than half of households with a smoker had adopted such rules.

The national prevalence of smokefree home rules increased from 43.0% during 1992–1993 to 83.0% during 2010–2011 (p<0.05) (Table). Prevalence ranged from 25.6% in Kentucky to 69.4% in Utah during 1992–1993, and from 69.4% in Kentucky to 93.6% in Utah during 2010–2011 (Figure).

Among households with no adult smokers, the national prevalence of smokefree home rules increased from 56.7% during 1992–1993 to 91.4% during 2010–2011 (p<0.05). Prevalence ranged from 39.2% in Kentucky to 82.8% in Utah during 1992–1993, and from 82.9% in West Virginia to 97.3% in Utah during 2010–2011.

Among households with at least one adult smoker, the national prevalence of smokefree home rules increased from 9.6% during 1992–1993 to 46.1% during 2010–2011 (p<0.05). Prevalence ranged from 3.6% in Kentucky to 20.9% in Utah during 1992–1993, and from 27.2% in West Virginia to 68.4% in Utah during 2010–2011.

## Discussion

The prevalence of smokefree home rules among U.S. households increased considerably over the past 2 decades, from 43.0% during 1992–1993 to 83.0% during 2010–2011. Making homes completely smokefree reduces SHS exposure among nonsmokers, particularly children, and can help adult smokers quit (1). Despite these benefits, millions of nonsmokers in the United States remain unprotected by smokefree home rules. To continue to increase the percentage of U.S. households that are smokefree, efforts are warranted to educate the public about the dangers of SHS exposure and to encourage adoption of smokefree home rules, particularly among persons living in states with lower prevalence of these rules. Additionally, efforts to implement smokefree policies in multiunit housing, where residents who have instituted smokefree home rules can still be exposed to SHS that enters their units from other units and shared areas where smoking occurs (6), would further protect nonsmokers from SHS exposure in their homes.

The increased prevalence of smokefree home rules observed nationally and across all states might be attributable to multiple factors, including the spread of state and local comprehensive smokefree laws covering public places and worksites, and declines in cigarette smoking prevalence (1,2). Additionally, the substantial increases in the prevalence of smokefree rules in households with at least one smoker and in households in states with high cigarette smoking rates might reflect changes in public attitudes about the social acceptability of smoking around nonsmokers (1,2). Comprehensive smokefree laws can stimulate the adoption of voluntary smokefree home rules and increase support for smokefree environments among both nonsmokers and smokers (1,7). As of April 2014, 26 states, the District of Columbia, and approximately 600 local municipalities had implemented comprehensive smokefree laws (3,4); almost half (49.2%) of U.S. residents are currently covered by comprehensive smokefree laws at the state or local level.§ Despite this progress, during 2007–2008, approximately 88 million U.S. residents aged ≥3 years were exposed to SHS, and disparities in exposure exist across subpopulations (5).

The findings in this report are subject to at least five limitations. First, smokefree rules were self-reported and not validated by an objective measure. However, parental reporting of smokefree home rules strongly correlates with child cotinine levels, suggesting that self-reports of smokefree home rules are accurate (8). Second, because the 2006–2007 and 2010–2011 TUS-CPS cycles were only administered to respondents aged ≥18 years, respondents aged 15–17 years who completed the 1992–1993 through 2003 TUS-CPS were excluded. However, excluding these persons did not have a significant impact on the findings; for example, during 1992–1993, national prevalence of smokefree home rules among respondents aged ≥18 years was 43.0%, compared with 43.2% among those aged ≥15 years. Third, members of households with discrepant reports of smokefree home rules were excluded; however, the percentage of excluded respondents was small and declined over time. Fourth, the study only assessed the presence of cigarette smokers in the home and might not have captured adults who smoked other tobacco products such as cigars. Finally, response rates for TUS-CPS have declined over time (from 72% during 1992–1993 to 62% during 2010–2011). Lower response rates can increase bias; however, the data were adjusted for nonresponse, and the estimates were comparable to other studies (9).

Although substantial progress has been made in increasing the prevalence of smokefree home rules, fewer than half of households with smokers have adopted such rules. This is concerning because nearly all nonsmokers who live with someone who smokes inside the home are exposed to SHS (5). Because 100% smokefree indoor environments are the only effective way to fully eliminate SHS exposure (1), efforts are warranted to educate the public about the dangers of SHS and to promote the adoption of smokefree home rules, particularly among subpopulations at greatest risk for exposure, such as those living in households with smokers, in states with lower prevalence of smokefree home rules, and in multiunit housing (1,2,5,10). Continued adoption of smokefree home rules, in concert with intensified implementation of comprehensive smokefree laws in indoor public places and worksites, can reduce nonsmokers’ exposure to this preventable health hazard (1,2,5).

## Figures and Tables

**FIGURE:**
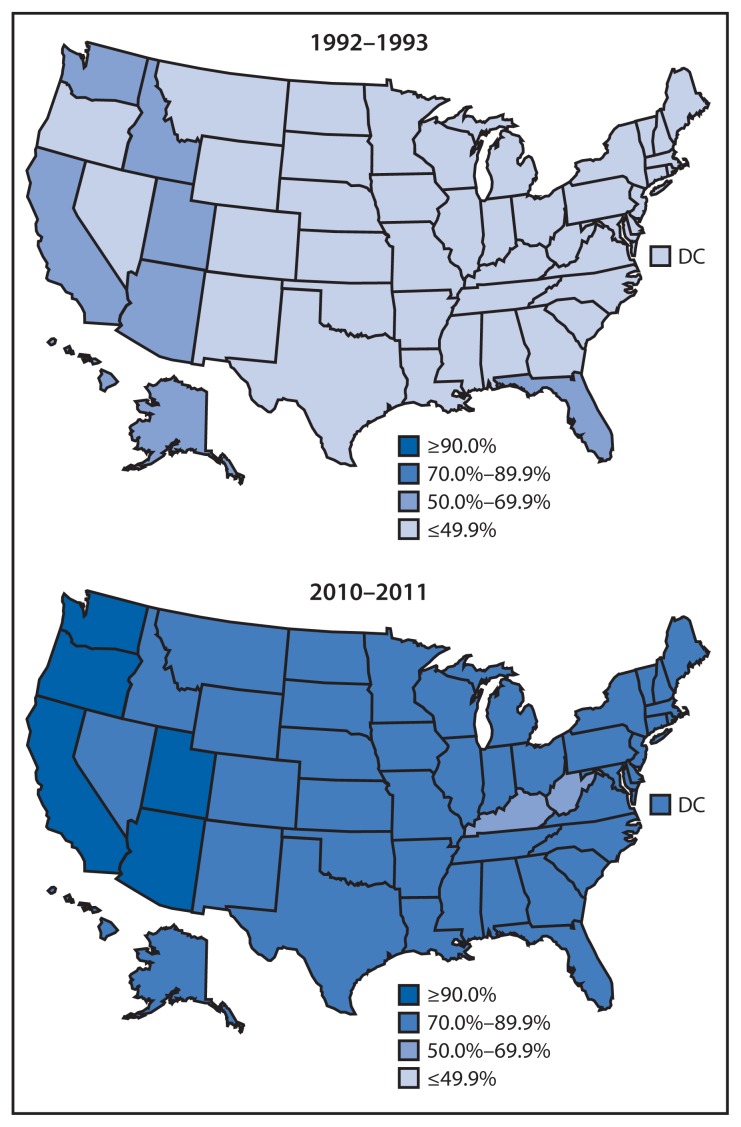
Percentage of households with a smokefree home rule,^*^ by state — Tobacco Use Supplement to the Current Population Survey, 1992–1993 and 2010–2011 ^*^ Households were considered to have a smokefree home rule if all adult respondents aged ≥18 years in the household reported that no one was allowed to smoke anywhere inside the home at any time.

**TABLE:** Percentage of households with a smokefree home rule,* by state, and whether an adult smoker lives in the household† — Tobacco Use Supplement to the Current Population Survey, 1992–1993 and 2010–2011

State	All households				Households with no adult smoker				Household with at least one adult smoker			
		
	1992–1993		2010–2011§		1992–1993		2010–2011§		1992–1993		2010–2011§	
					
	%	(95% CI)	%	(95% CI)	%	(95% CI)	%	(95% CI)	%	(95% CI)	%	(95% CI)
Alabama	38.7	(34.0–43.4)	80.9	(77.5–84.3)	54.1	(48.4–59.8)	91.3	(88.9–93.7)	6.7	(5.1–8.3)	38.4	(30.1–46.6)
Alaska	50.8	(46.9–54.7)	85.6	(82.3–88.8)	68.0	(63.8–72.1)	94.7	(93.1–96.3)	14.1	(8.3–19.8)	56.5	(48.9–64.0)
Arizona	54.1	(50.6–57.5)	91.0	(89.2–92.8)	68.2	(63.4–73.0)	96.4	(95.3–97.5)	17.2	(14.6–19.8)	64.8	(57.9–71.7)
Arkansas	33.1	(29.9–36.2)	73.1	(68.7–77.5)	46.7	(42.3–51.1)	85.5	(82.1–89.0)	5.3	(3.3–7.3)	35.9	(29.0–42.9)
California	59.0	(57.3–60.7)	91.5	(90.8–92.2)	71.6	(70.1–73.1)	94.9	(94.3–95.5)	19.0	(16.6–21.3)	67.9	(64.8–71.0)
Colorado	47.8	(44.8–50.8)	87.4	(85.4–89.4)	62.9	(59.3–66.6)	93.3	(91.8–94.7)	10.2	(6.6–13.8)	55.6	(49.8–61.4)
Connecticut	44.7	(42.2–47.2)	84.6	(82.8–86.3)	58.4	(54.6–62.3)	92.5	(91.1–93.8)	11.7	(8.8–14.7)	47.5	(41.9–53.0)
Delaware	40.0	(36.7–43.3)	80.4	(78.0–82.7)	52.2	(48.8–55.5)	90.2	(88.0–92.3)	9.9	(5.2–14.6)	39.1	(33.5–44.8)
DC	41.3	(37.6–43.3)	80.7	(78.4–83.0)	52.8	(48.5–57.0)	89.3	(87.3–91.2)	5.5	(1.6–9.5)	31.7	(25.7–37.7)
Florida	50.1	(48.2–51.9)	88.3	(87.1–89.4)	64.8	(62.8–66.7)	94.5	(93.7–95.4)	13.2	(10.6–15.7)	57.1	(53.3–60.9)
Georgia	41.4	(38.4–44.3)	84.9	(82.9–86.8)	55.1	(51.2–59.0)	91.5	(89.6–93.3)	7.9	(4.9–10.9)	51.9	(47.0–56.7)
Hawaii	51.2	(47.1–55.4)	85.1	(82.7–87.5)	64.6	(59.5–69.7)	89.9	(87.6–92.2)	12.7	(8.6–16.7)	57.3	(48.5–66.1)
Idaho	50.0	(45.1–54.9)	88.6	(87.0–90.2)	66.1	(60.5–71.7)	95.1	(93.7–96.4)	11.5	(8.9–14.1)	61.6	(55.8–67.3)
Illinois	38.5	(35.6–41.5)	79.2	(77.7–80.7)	51.3	(48.3–54.2)	89.0	(87.6–90.3)	7.2	(4.9–9.5)	38.1	(33.7–42.5)
Indiana	33.9	(30.9–36.9)	73.9	(71.0–76.9)	47.6	(43.4–51.8)	86.3	(83.9–88.7)	7.8	(4.5–11.1)	31.4	(25.6–37.2)
Iowa	35.9	(33.1–38.8)	78.4	(76.8–80.0)	48.0	(44.4–51.6)	89.4	(87.8–91.0)	5.6	(3.7–7.4)	41.4	(37.2–45.5)
Kansas	39.6	(36.0–43.2)	81.1	(78.1–84.1)	54.9	(51.6–58.2)	91.8	(90.1–93.5)	4.9	(3.2–6.7)	43.1	(37.7–48.4)
Kentucky	25.6	(21.4–29.8)	69.4	(66.9–71.8)	39.2	(33.3–45.0)	84.5	(82.5–86.6)	3.6	(2.3–5.0)	29.3	(24.8–33.8)
Louisiana	37.0	(33.3–40.7)	82.5	(79.7–85.2)	47.8	(44.1–51.5)	92.0	(90.1–93.9)	11.6	(7.1–16.1)	45.6	(39.6–51.6)
Maine	39.5	(34.6–44.4)	82.0	(79.8–84.1)	57.5	(51.7–63.4)	90.6	(89.0–92.2)	8.1	(5.1–11.1)	50.5	(45.7–55.3)
Maryland	42.4	(38.9–45.8)	84.3	(82.5–86.1)	56.7	(53.2–60.2)	90.6	(88.9–92.3)	6.3	(3.1–9.5)	48.9	(43.4–54.4)
Massachusetts	40.2	(38.1–42.3)	84.1	(81.9–86.3)	51.2	(49.1–53.2)	91.8	(90.2–93.5)	10.0	(7.8–12.2)	42.2	(35.5–49.0)
Michigan	35.0	(33.1–36.9)	76.3	(74.4–78.2)	49.1	(46.8–51.3)	87.2	(85.6–88.9)	6.1	(4.9–7.3)	36.0	(31.4–40.5)
Minnesota	39.6	(37.8–41.4)	84.2	(82.9–85.6)	53.8	(50.9–56.6)	92.8	(91.8–93.8)	7.8	(5.2–10.3)	48.9	(44.1–53.8)
Mississippi	40.9	(37.1–44.7)	80.2	(77.3–83.2)	53.9	(49.1–58.6)	88.8	(85.9–91.6)	9.1	(6.3–12.0)	47.4	(38.9–55.9)
Missouri	34.1	(30.1–38.1)	74.1	(71.1–77.0)	46.0	(41.7–50.4)	87.1	(84.8–89.4)	7.6	(4.4–10.8)	36.0	(30.3–41.7)
Montana	42.8	(38.8–46.7)	82.8	(79.9–85.7)	56.8	(53.1–60.5)	91.5	(88.8–94.2)	7.4	(5.3–9.4)	49.7	(42.7–56.7)
Nebraska	40.0	(36.3–43.7)	82.3	(79.9–85.7)	52.2	(47.6–56.8)	90.8	(89.2–92.3)	8.6	(6.7–10.6)	49.2	(43.6–54.9)
Nevada	45.5	(42.5–48.4)	86.5	(84.6–88.4)	62.5	(59.4–65.6)	94.3	(92.9–95.7)	10.3	(6.8–13.7)	55.1	(47.9–62.4)
New Hampshire	38.3	(34.7–42.0)	83.5	(81.7–85.4)	51.5	(47.4–55.6)	92.5	(91.0–93.9)	7.3	(3.9–10.8)	44.4	(39.1–49.8)
New Jersey	45.5	(43.2–47.7)	86.1	(84.3–88.0)	58.3	(56.3–60.3)	92.7	(91.4–94.0)	10.1	(8.5–11.7)	47.5	(40.8–54.2)
New Mexico	45.4	(40.8–50.0)	84.4	(82.2–86.6)	58.8	(53.1–64.6)	90.9	(88.7–93.2)	11.4	(5.3–17.5)	54.7	(45.0–64.5)
New York	41.4	(39.6–43.2)	81.2	(79.8–82.7)	53.7	(52.2–55.2)	89.8	(88.6–90.9)	8.1	(6.2–10.0)	36.5	(32.8–40.2)
North Carolina	34.1	(32.3–35.9)	79.4	(77.1–81.8)	46.2	(44.1–48.4)	90.2	(88.5–91.8)	8.6	(7.2–10.0)	36.7	(31.0–42.5)
North Dakota	40.9	(36.8–45.0)	81.2	(78.1–75.7)	53.0	(48.4–57.6)	90.6	(89.0–92.2)	8.3	(6.1–10.5)	47.7	(41.9–53.4)
Ohio	35.0	(33.5–36.5)	73.7	(71.8–75.7)	47.9	(46.0–49.8)	86.4	(84.8–88.1)	6.0	(4.7–7.2)	34.3	(30.3–38.3)
Oklahoma	39.1	(35.0–43.1)	76.4	(73.5–79.4)	55.2	(50.6–59.7)	90.3	(88.3–92.3)	6.0	(4.6–7.5)	40.5	(32.8–48.2)
Oregon	49.8	(45.8–53.8)	90.8	(88.9–92.8)	64.5	(60.3–68.6)	95.9	(94.5–97.2)	13.1	(7.9–18.4)	65.6	(58.4–72.9)
Pennsylvania	39.6	(37.9–41.3)	78.5	(77.0–80.0)	52.7	(50.8–54.5)	88.3	(86.9–89.8)	7.9	(6.3–9.6)	39.9	(36.0–43.9)
Rhode Island	38.9	(34.1–43.8)	79.4	(77.1–81.6)	52.6	(46.7–58.5)	90.1	(88.3–91.9)	6.6	(3.8–9.4)	37.5	(31.8–43.3)
South Carolina	39.9	(37.3–42.5)	78.0	(75.4–80.7)	54.3	(51.0–57.7)	88.7	(85.6–91.9)	7.4	(5.4–9.4)	33.1	(26.5–39.7)
South Dakota	36.7	(34.1–39.2)	80.8	(78.8–82.8)	50.0	(47.1–52.9)	89.8	(87.9–91.6)	5.2	(3.4–7.1)	52.5	(47.4–57.6)
Tennessee	33.9	(30.5–37.3)	75.0	(72.1–77.9)	48.8	(44.6–53.1)	87.7	(84.9–90.5)	4.6	(3.6–5.5)	35.8	(31.2–40.3)
Texas	46.3	(43.6–49.0)	85.1	(83.9–86.3)	60.3	(57.6–63.0)	92.5	(91.7–93.4)	10.6	(8.5–12.6)	51.7	(47.9–55.6)
Utah	69.4	(65.5–73.2)	93.6	(92.0–95.2)	82.8	(80.4–85.2)	97.3	(96.2–98.4)	20.9	(13.1–28.7)	68.4	(59.9–76.8)
Vermont	39.0	(35.3–42.7)	85.0	(83.1–86.9)	54.6	(50.3–58.9)	92.1	(90.6–93.6)	8.3	(4.6–11.9)	56.1	(50.1–62.0)
Virginia	39.0	(35.9–42.1)	85.6	(82.6–88.5)	53.8	(49.5–58.0)	93.2	(91.5–94.9)	7.4	(5.1–9.7)	46.1	(39.6–52.6)
Washington	54.3	(50.4–58.3)	90.7	(89.2–92.2)	69.5	(65.0–74.0)	95.2	(93.9–96.4)	16.9	(13.4–20.4)	70.2	(65.8–74.6)
West Virginia	27.9	(24.0–31.8)	69.0	(65.8–72.2)	41.8	(36.9–46.7)	82.9	(79.8–85.9)	4.0	(2.8–5.2)	27.2	(22.3–32.1)
Wisconsin	36.5	(33.3–39.6)	83.1	(80.7–85.5)	50.4	(47.4–53.3)	91.4	(90.0–92.8)	5.9	(4.3–7.6)	49.4	(42.9–55.9)
Wyoming	38.5	(34.5–42.4)	78.8	(75.3–82.2)	52.8	(48.6–57.1)	90.3	(87.9–92.6)	6.2	(4.1–8.2)	41.1	(34.4–47.9)
**Overall**	**43.0**	**(42.1–43.9)**	**83.0**	**(82.7–83.4)**	**56.7**	**(55.9–57.5)**	**91.4**	**(91.1–91.6)**	**9.6**	**(8.8–10.4)**	**46.1**	**(45.2–47.0)**

**Abbreviations:** CI = confidence interval; DC = District of Columbia.

* Households were considered to have a smokefree home rule if all adult respondents aged ≥18 years in the household reported that no one was allowed to smoke anywhere inside the home at any time.

† Households were considered to have at least one adult smoker if at least one adult resident aged ≥18 years reported that they had smoked ≥100 cigarettes in their lifetime and smoked “every day” or “some days” at the time of survey.

§ Statistically significant increases were observed from 1992–1993 to 2010–2011, overall and in all states (p<0.05).

## Footnotes

* Additional information available at http://www.census.gov/prod/2000pubs/tp63.pdf.

† Additional information available at http://riskfactor.cancer.gov/studies/tus-cps/info.html.

§ Additional information available at http://www.no-smoke.org/pdf/SummaryUSPopList.pdf.
